# Aurora A regulates expression of AR-V7 in models of castrate resistant prostate cancer

**DOI:** 10.1038/srep40957

**Published:** 2017-02-16

**Authors:** Dominic Jones, Martin Noble, Steve R. Wedge, Craig N. Robson, Luke Gaughan

**Affiliations:** 1 Northern Institute for Cancer Research, Newcastle University, Paul O’Gorman Building, Framlington Place, Newcastle upon Tyne, Ne2 4HH, UK

## Abstract

Androgen receptor variants (AR-Vs) provide a mechanism of therapy evasion in castrate-resistant prostate cancer (CRPC), yet mechanisms of regulation remain largely unknown. Here we investigate the role of Aurora A kinase on AR-Vs in models of CRPC and show depletion of Aurora A reduces AR-V target gene expression. Importantly, knockdown of Aurora A reconfigures splicing of AR pre-mRNA to discriminately down-regulate synthesis of AR-V transcripts, including AR-V7, without effecting full-length AR mRNA; and as a consequence, AR-V-driven proliferation and survival of CRPC cells is markedly reduced. Critically, these effects are reproduced by Aurora A inhibition. We show that Aurora A levels increase in advanced disease and *AURKA* is an AR-V target gene demonstrating a positive feedback mechanism of androgenic signalling in CRPC. In all, our data suggests that Aurora A plays a pivotal role in regulation of AR-V7 expression and represents a new therapeutic target in CRPC.

Prostate cancer (PC) is the most frequently diagnosed cancer in men in the Western world and is the second leading cause of male-related cancer deaths. Current therapeutic strategies exploit the androgen-dependency of tumour growth by inactivating the androgen receptor (AR) with anti-androgens and androgen-deprivation therapies (ADT)^1^,^2^. Although ADT is initially efficacious, most patients often relapse with a more aggressive form of the cancer, termed castrate resistant prostate cancer (CRPC) which remains challenging to treat and unfortunately remains largely fatal^3^. Development of second-generation AR targeting agents such as enzalutamide and abiraterone have provided some benefit for patients with CRPC^4^,^5^,^6^, but the emergence of resistance has limited their clinical success^7^. The AR, a nuclear hormone receptor transcription factor, activates the androgenic signalling axis in response to binding testosterone and dihydrotestosterone to promote prostate growth and transformation. Importantly, AR signalling remains active in most patients with advanced disease even in the presence of anti-androgenic therapies, and therefore this pathway remains a prominent target in CRPC^8^.

The transcriptional activity of the AR is tightly regulated by a plethora of co-regulatory proteins which control the response to androgenic stimulation by a number of mechanisms, including post translational modifications^9^. Phosphorylation of the AR by a number of protein kinases has been extensively investigated and demonstrated as an important mechanism of modulating receptor activity^9^. One particular kinase shown to phosphorylate AR, Aurora A kinase (hereby referred to as Aurora A), has been shown to be a co-activator of the AR; phosphorylating two residues within the N-terminal domain of the receptor. Depletion of Aurora A leads to cell growth arrest and apoptosis, whilst overexpression results in an increase in AR target gene expression^10^.

A number of aberrations to AR signalling have been characterised as mechanisms of resistance to first line ADT including *AR* gene amplification^11^ and mutation^12^ which enables retention of AR function, even in castrate conditions. Recently, the emergence of AR variants (AR-Vs) has been identified as a novel mechanism of therapy resistance and tumour progression. These variants lack the conventional C-terminal ligand binding domain (LBD), but retain the transcriptionally potent N-terminal domain (NTD) and DNA-binding domain of the receptor^13^,^14^. One clinically relevant receptor variant, AR-V7, has been shown to bind DNA and drive androgenic signalling in castrate conditions, and remains refractory to current AR targeting agents^15^. Studies have suggested that AR-Vs are expressed in 50–60% of CRPC patients^16^ and therefore represent a potential common mechanism of therapy resistance.

How these AR-Vs are regulated remains largely understudied; particularly their dependency on co-regulatory proteins. In this study, we show that Aurora A depletion discriminately reduces AR-V levels in CWR22R*v*1 cells by alternate splicing mechanisms which subsequently reduces AR-V target gene transcription including *TMPRSS2* and *UBE2C*. Moreover, depletion of Aurora A reduces cell growth and survival by attenuating AR-dependent cell proliferation. Consistent with a role of Aurora A kinase activity in AR-V regulation, small molecule inhibition of Aurora A reduces AR-V levels, AR-V target gene expression and cell proliferation. In all, our data suggests multiple non-mitotic roles for Aurora A in PC progression and resistance to first-line therapies, and therefore may represent a viable therapeutic target in the treatment of advanced disease.

## Results

### AR-Vs regulate cell cycle gene expression

**Figure 1:**
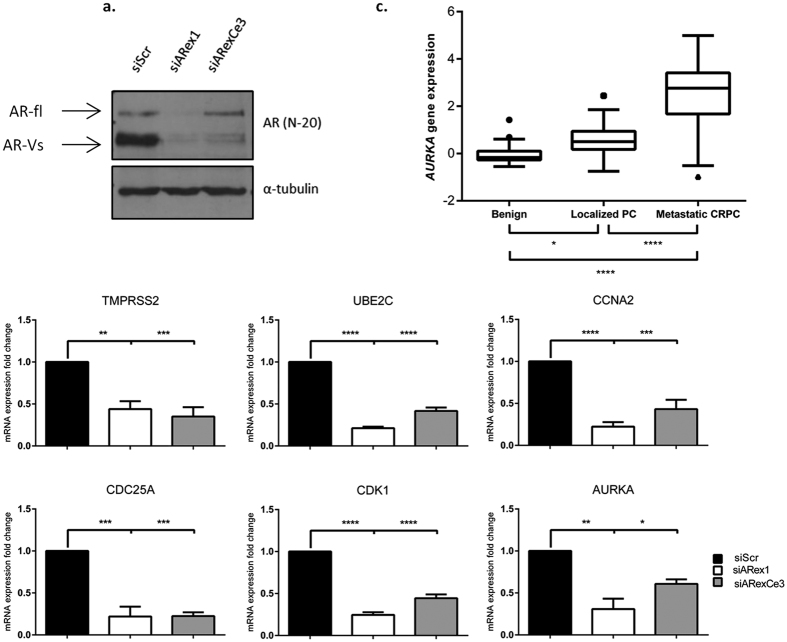
AR-Vs regulate gene expression in CWR22R*v*1 cells. (**a**) FL-AR (siARex1) and AR-V7 (siARexCe3) species were reduced by siRNA knockdown for 72 hours in CWR22R*v*1 cells grown in steroid-depleted conditions prior to western analysis using an anti-AR antibody. (**b**) Expression of *TMPRSS2, UBE2C, CCNA2, CDC25A, CDK1* and *AURKA* was measured by quantitative PCR in samples described in (**a**). Data is represented as fold-change of siScr experimental arm. Data represents three independent experiments ± SEM. P-values were determined by one-way ANOVA followed by a Bonferroni multiple comparison test (*, **, ***and ****denote p-values < 0.05, <0.01, <0.001, <0.0001, respectively). (**c**) *AURKA* gene expression was measured in benign prostate (n = 28), localized PC (n = 59) and metastatic CRPC (n = 35) from the Grasso dataset^19^. Outliers are shown as ●, ■ and ▲, respectively (p = 0.0130, p < 0.0001, p, 0.0001).

Androgen receptor variants (AR-Vs) have been identified to confer resistance to current AR-targeting therapies. The clinically-relevant AR-V, AR-V7, has previously been shown to be constitutively associated with chromatin in CWR22Rv1 cells and promotes cell proliferation in castrate conditions^17^. Here we show that depletion of all AR species in CWR22Rv1 cells grown in conditions where only AR-Vs are active^17^ using an *AR* exon 1-targeting siRNA (siARex1) (Fig. 1a) significantly reduced the expression of a plethora of important cell cycle regulatory genes including *UBE2C, CCNA2, CDC25A, CDK1* and *AURKA*, in addition to the canonical AR target gene *TMPRSS2,* suggesting that AR-V signalling is critical for cell cycle control (Fig. 1b). Importantly, depletion of AR-Vs (siARexCe3) phenocopied that of total AR knockdown resulting in significant reductions to *TMPRSS2, UBE2C, CCNA2, CDC25A, CDK1* and *AURKA* expression (Fig. 1b). Critically, this finding is consistent with our previously published micro-array data in which cells grown in steroid-depleted conditions and enzalutamide demonstrated robust down-regulation of AURKA mRNA in response to AR-V knockdown^17^, implicating an important role for receptor variants maintaining *AURKA* expression in the absence of FL-AR signalling.

Interestingly, Aurora A kinase (*AURKA* gene product) has been previously shown to modulate full-length AR activity and thus represents a potential mechanism of positive feedback that may enhance AR signalling. Although Aurora A kinase expression has been shown to be increased in prostatic intraepithelial neoplasia lesions and localised hormone naïve prostate tumours compared to non-neoplastic samples^18^, expression in CRPC is largely unknown. Using the Grasso dataset^19^, *AURKA* expression in benign prostate tissue, localised PC and metastatic CRPC was assessed. We observed a significant increase in *AURKA* expression in CRPC (n = 35) compared to primary localised PC (n = 59) and benign (n = 28) samples (p = <0.0001 and p = <0.0001, respectively) with a significant increase between benign and localise PC samples (p = 0.0130) (Fig. 1c) suggesting a role of Aurora A in prostate cancer progression.

### Aurora A depletion reduces AR target gene expression

**Figure 2:**
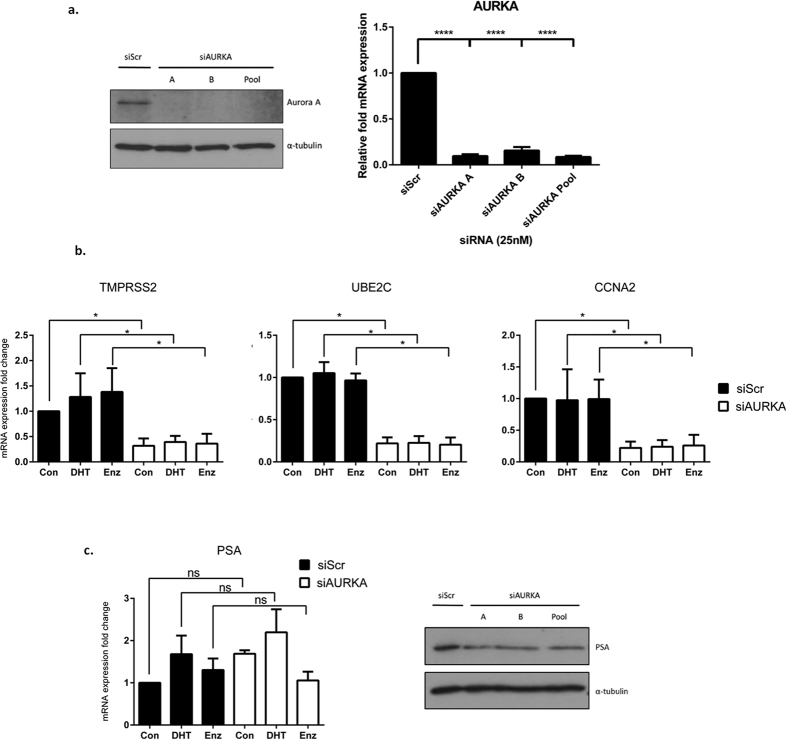
Aurora A depletion reduces AR-V target gene expression. (**a**) Aurora A protein (left panel) and mRNA levels (right panel) were analysed after 72 hour siRNA depletion of Aurora A in CWR22R*v*1 cells grown in steroid-depleted conditions. Data represents three independent experiments ± SEM. P-values were determined by one-way ANOVA followed by a Bonferroni multiple comparison test (****denotes p-values < 0.0001). (**b**) Impact of Aurora A knockdown (siAURKA A) on *TMPRSS2, UBE2C* and *CCNA2* expression in CWR22R*v*1 cells grown in steroid-depleted conditions +/− 10 nM DHT or 10 μM enzalutamide. Data represents three independent experiments ± SEM. P-values were determined by Turkey’s multiple comparison test following two-way ANOVA (*denotes p-values < 0.05). (**c**) Effect of Aurora A knockdown on PSA mRNA (left panel) in CWR22Rv1 cells grown in steroid-depleted conditions +/− 10 nM DHT or 10 μM enzalutamide was analysed by quantitative PCR and western analysis. ns denotes a non-significant p-value. Representative western blot analysis of PSA protein levels (right panel) were measured from CWR22R*v*1 cells grown in steroid-depleted conditions following aurora A knockdown using multiple siRNAs.

Aurora A directly phosphorylates the N-terminus of the FL-AR in a ligand-independent manner to promote binding of the receptor to *cis-*regulatory elements of target genes and enhance transcription^10^ (Supplementary Figure S1). To examine a role for Aurora A in AR-V regulation, we firstly assessed the impact of Aurora A depletion on expression of the AR target genes *PSA, TMPRSS2, UBE2C* and *CCNA2*^20^ in CWR22Rv1 cells grown in steroid-depleted media supplemented with either 10 nM DHT or 10 μM enzalutamide. Using two individual and pooled siRNAs to reduce Aurora A (Fig. 2a), expression of *TMPRSS2, UBE2C* and *CCNA2* were robustly down-regulated in the presence of DHT and enzalutamide supporting the concept that Aurora A is important for the activity of FL and splice variant forms of the receptor (Fig. 2b and Supplementary Figure S2). Interestingly, PSA mRNA was unaffected by Aurora A depletion, but demonstrated robust down-regulation at the protein level (Fig. 2c).

**Figure 3:**
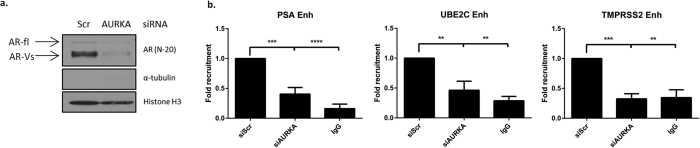
Aurora A depletion reduces AR-V deposition at androgen response elements. (**a**) Chromatin fractionation was performed in CWR22R*v*1 cells transfected with siAURKA A or control siScr. Resultant samples were subjected to western analysis using anti-AR (N-20), -α-tubulin (cytoplasmic fraction) and –histone H3 (chromatin fraction) antibodies. (**b**) Chromatin immunoprecipitation was performed on CWR22R*v*1 cells grown in steroid-depleted conditions transfected with siAURKA or siScr using an anti-AR antibody. Data is represented as a fold change of siScr. Data represents three independent experiments ± SEM. Statistical significance was measured by one-way ANOVA followed by a Bonferroni multiple comparison test (***denotes p-values < 0.001).

We next assessed the role of Aurora A in modulating AR-V chromatin binding. Consistent with our previous report^17^ we show that AR-Vs are constitutively bound to chromatin in CWR22Rv1 cells in the absence of androgens and is in marked contrast to the full length receptor which is not enriched in this fraction (Fig. 3a). Importantly, Aurora A depletion perturbed global AR-V binding to chromatin (Fig. 3a). Moreover, using chromatin immunoprecipitation (ChIP) incorporating an anti-AR antibody, we demonstrate that knockdown of Aurora A significantly reduces discriminate AR-V binding to cis-regulatory elements of *PSA, TMPRSS2* and *UBE2C* genes in castrate conditions (Fig. 3b).

### Aurora A knockdown reduces proliferation of PC models

**Figure 4:**
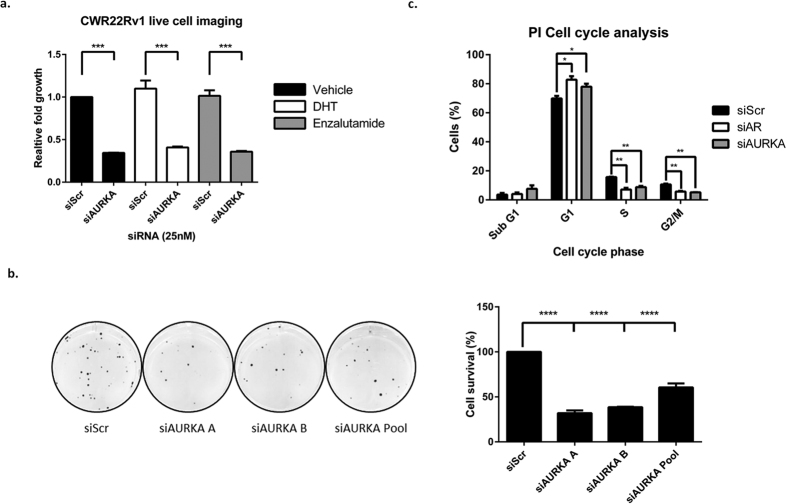
Depletion of Aurora A reduces cell proliferation and survival. (**a**) CWR22R*v*1 cells were transiently transfected with siScr or siAURKA A and grown in steroid-depleted conditions +/− 10 nM DHT or 10 μM enzalutamide in the IncucyteZoom live cell imager. Cell confluence was measured every 6 hours over a 96 hour period and % confluence data analysed. Data is represented as a fold change of siScr vehicle. Data represents three independent experiments ± SEM. P-values were determined by two-way ANOVA using Turkey’s multiple comparison test. (**and ****denote p-values < 0.01 and <0.0001, respectively). (**b**) Impact on cell survival of CWR22R*v*1 cells depleted of Aurora A was measured using colony forming assays to measure survival after 12 days of siRNA-mediated Aurora A knockdown compared to a scrambled control arm, set to 100. One-way ANOVA followed by a Bonferroni multiple comparison test was performed to test statistical significance (****denotes p-value < 0.0001). (**c**) Cell cycle analysis of CWR22R*v*1 grown in steroid-depleted conditions was measured by propidium iodide (PI) flow cytometry following AR or Aurora A knockdown for 72 hours. Data represents three independent experiments ± SEM. P-values were determined by one-way ANOVA followed by a Bonferroni multiple comparison test (*and **denote p-values < 0.05 and <0.01, respectively).

Live cell imaging was next applied to examine the impact of Aurora A depletion on CWR22R*v*1 cell proliferation. As expected, both DHT and enzalutamide failed to impact on cell growth and is consistent with previous findings showing the pro-proliferative role of AR-Vs (Fig. 4a)^14^. Importantly, cell proliferation in castrate conditions was markedly reduced by depletion of Aurora A compared to siRNA control (siScr). This was further supported by the demonstration that Aurora A knockdown reduced the colony forming ability of CWR22Rv1 cells indicating an important role of the kinase in PC cell proliferation and survival (Fig. 4b). Given the well characterised roles of Aurora A in regulating spindle formation during early mitosis, and more recently as a FL-AR co-regulator, it was pertinent to profile the impact of Aurora A knockdown on cell cycle in the CWR22Rv1 cell line. As shown in Fig. 4c, depletion of Aurora A significantly increases the proportion of cells in G1 at the expense of S and G2/M phases of the cell cycle and mimics that of combined FL-AR and AR-V knockdown. This suggests that Aurora A regulates proliferation of CWR22Rv1 cells principally by co-activating the AR to drive cells into S phase; a dependency that masks any potential impact on the later mitotic phase of the cell cycle. In support of this, we show that knockdown of Aurora A in AR-negative PC-3 cells causes accumulation in G2/M phase of the cell cycle, while in AR-positive LNCaP cells, G2/M is modestly reduced (Supplementary Figure 3).

### Aurora A depletion reduces AR-V expression

**Figure 5:**
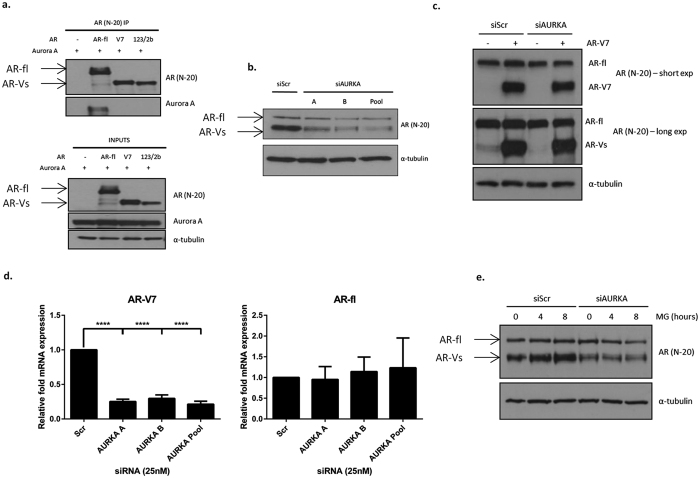
Aurora A regulates AR-V expression. (**a**) Co-immunoprecipitation was carried out using the AR (N-20) antibody on HEK293 cells overexpressing FL-AR, AR-V7 or AR-123/2b and Aurora A followed by subsequent western analysis using anti-AR (N-20) and –Aurora A antibodies. Input samples were also subjected to western blot analysis (bottom panel). (**b**) Impact of Aurora A depletion on AR levels was measured by western blot analysis of CWR22R*v*1 cells transiently transfected with Aurora A-targeting siRNA. (**c**) LNCaP cells were transiently transfected with siAURKA and incubated for 6 hours prior to transduction with lentivirus expressing AR-V7 for 72 hours. Resultant lysates were subjected to western blot analysis. (**d**) Parallel samples of those described in (**b**) were subjected to quantitative-PCR to measure AR-V7 and FL-AR mRNA expression following Aurora A depletion. Data is represented as fold-change of siScr. Data represents three independent experiments ± SEM. P-values were determined by one-way ANOVA followed by a Bonferroni multiple comparison test (****denotes p-value < 0.001). (**e**) CWR22R*v*1 cells were transiently transfected with siAURKA or siScr for 72 hours with the addition of 20 μM MG132 (MG) for the final 4 or 8 hours before harvest. AR levels were measured by western blot analysis using the AR (N-20) antibody.

Given the Aurora A phosphorylation sites are located in the AR NTD, we next investigated whether, like FL-AR, AR-Vs interact with Aurora A in PC cells. Aurora A and either FL-AR, AR-V7 and the clinically-relevant AR-123/2b, were co-transfected into PC-3 cells for 48 hours prior to immunoprecipitation using an NTD-binding anti-AR antibody. As shown in Fig. 5a, probing FL-AR immunoprecipitates with an anti-Aurora A antibody demonstrated interaction between the two proteins and is consistent with a previous report^10^. Surprisingly, however, no interaction was detected between the distinct AR-Vs and Aurora A even though robust effects of kinase depletion on AR-V transcriptional activity and chromatin binding had been demonstrated (Figs 2 and 3). We therefore hypothesised that Aurora A may regulate AR-V activity independently of direct interaction and phosphorylation in CWR22Rv1 cells. Upon examination of full-length and variant isoform protein levels in cells depleted of Aurora A, we observed markedly reduced AR-Vs while FL-AR remained largely unchanged (Fig. 5b). This specific reduction of AR-V levels upon depletion of Aurora A was also evident in VCaP cells, a CRPC model that expresses AR-V7, albeit considerably lower than that observed in CWR22R*v*1 cells (Supplementary Figure S4). To investigate whether differences in FL-AR and AR-V7 protein levels was a result of altered stability of AR-Vs or aberrant transcription of the *AR* gene, we examined protein levels of ectopically-expressed AR-V7 in LNCaP cells depleted of Aurora A. As shown in Fig. 5c, ectopic AR-V7 was not sensitive to Aurora A knockdown, supporting the concept that Aurora A does not control AR-V stability, but instead regulates AR-V mRNA processing. Consistent with this, the low abundance AR-Vs in LNCaP cells (as reported in refs 17 and 21) demonstrate sensitivity to Aurora A depletion, while FL-AR levels remain unchanged akin to that observed in CWR22Rv1 cells. To further validate our observations, addition of the proteosomal inhibitor MG132 failed to rescue AR-V protein levels in cells depleted of Aurora A (Fig. 5d). Importantly, knockdown of the kinase in CWR22Rv1 cells dramatically decreased AR-V7 and AR-123/2b mRNA without affecting FL-AR mRNA levels (Fig. 5e and Supplementary Figure S5) and is in agreement with our western analysis. Taken together, these results suggest that the effect of Aurora A knockdown on attenuated AR-V-mediated transcription and chromatin enrichment is a consequence of compromised AR-V mRNA processing.

### Aurora A modulates splicing of AR transcripts

**Figure 6:**
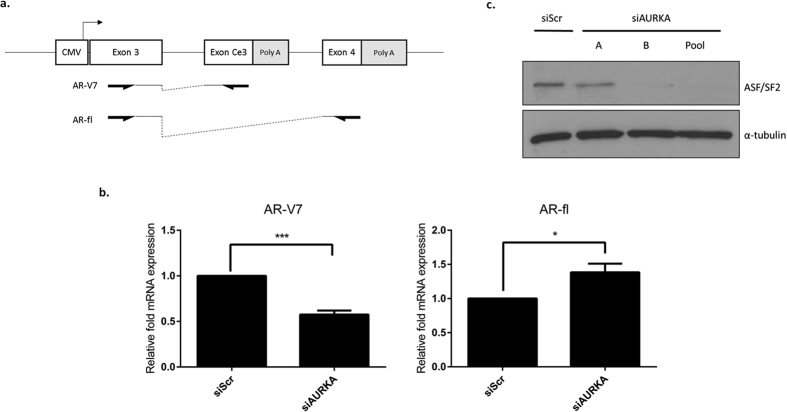
Inclusion of AR cryptic exons is reduced upon Aurora A depletion. (**a**) Diagrammatic representation of the AR-V7 minigene and primer binding sites specific to detecting transcripts representative of AR-V7 and FL-AR. (**b**) AR-V7 and FL-AR transcripts were measured using qPCR from PC-3 cells transfected with the AR-V7 minigene and depleted of Aurora A compared to the siScr control. Data represents three independent experiments ± SEM. P-values were determined by unpaired two-tailed Student’s t test (*and ***denote p-values < 0.05 and <0.01, respectively). (**c**) Levels of splicing factor ASF/SF2 were measured by western blot analysis in CWR22R*v*1 cells subjected to Aurora A depletion.

The discriminate effect of Aurora A depletion on AR-V mRNA levels suggested a role for the kinase in regulating alternative splicing of the *AR* gene. To assess this, we utilised a constitutive CMV-driven mini-gene reporter construct encompassing cryptic exon 3 (Ce3), specific to AR-V7, flanked by canonical exons 3 and 4 and their adjoining intronic sequences (Fig. 6a). To investigate the effect of Aurora A on alternative splicing of AR exons, the minigene construct was transfected into the AR negative cell line PC-3 that was subsequently depleted of Aurora A by siRNA. Using primers to specifically detect transcripts containing either exon Ce3 or exon 4, the role of Aurora A on cryptic exon inclusion was measured. As shown in Fig. 6b, inclusion of Ce3, and hence generation of AR-V7 transcripts, was reduced by approximately 50% in response to Aurora A knockdown, whilst mRNAs representing FL-AR were slightly elevated (Fig. 6b) supporting a role of Aurora A in regulating AR-V7 processing in CWR22Rv1.

Serine/arginine-rich splicing factor 1 (ASF/SF2) is one of a limited number of proteins that has previously been shown to modulate splicing of AR pre-mRNA and is required for the inclusion of exon Ce3^21^. Therefore, we sought to establish if Aurora A has downstream effects on ASF/SF2 and subsequently AR-V7 mRNA generation. Interestingly, levels of ASF/SF2 are markedly reduced upon Aurora A depletion in CWR22R*v*1 (Fig. 6c) suggesting a mechanism of control of AR-V expression by indirect regulation of key splicing factors by the kinase.

### Aurora A inhibition reduces AR activity and proliferation of PC

**Figure 7:**
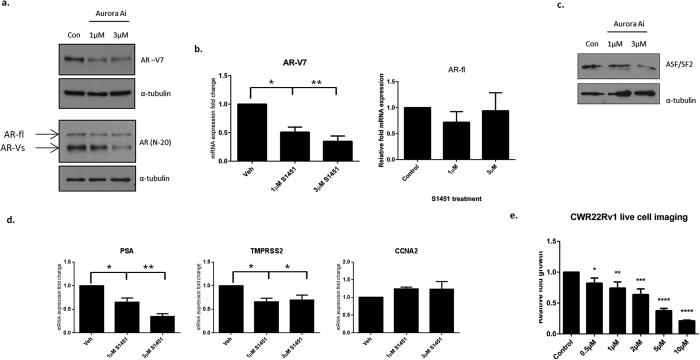
Inhibition of Aurora A reduces AR-V7 levels, reduces AR target gene expression and reduces proliferation in CWR22R*v*1 cells. (**a–d**) CWR22R*v*1 cells were grown in steroid-depleted conditions and treated with 1 or 3 μM Aurora A inhibitor (S1451) for 24 hours. AR protein levels (**a**) and transcripts (**b**) were measured by respective western blot and quantitative PCR analyses. qPCR data is represented as fold-change of Vehicle (Veh). Data represents three independent experiments ± SEM. P-values were determined by one-way ANOVA followed by a Bonferroni multiple comparison test (*and **denote p-values < 0.05 and <0.01, respectively). (**c**) ASF/SF2 levels were measured by western blot analysis following Aurora A inhibition. (**d**) *PSA, TMPRSS2* and *CCNA2* expression was measured by quantitative-PCR. qPCR data is represented as fold-change of Vehicle (Veh). Data represents three independent experiments ± SEM. P-values were determined by one-way ANOVA followed by a Bonferroni multiple comparison test (*and **denote p-values < 0.05 and <0.01, respectively). (**e**) Effects of Aurora A inhibition of CWR22R*v*1 cell proliferation was measured by subjecting CWR22R*v*1 cells grown in steroid-depleted conditions to increasing doses of the Aurora A inhibitor (0–10 μM). Proliferation was measured using the IncucyteZoom live cell imager and normalised to control. Data represents three independent experiments ± SEM. P-values were determined one-way ANOVA followed by a Bonferroni multiple comparison test (*, **, ***and ****denote p-values < 0.05, <0.01, <0.001 and <0.0001, respectively).

Development and utilisation of Aurora A inhibitors for the treatment of various cancers have shown success in several pre-clinical models. We therefore sought to establish whether the effects of Aurora A depletion in CRPC can be phenocopied by inhibition of Aurora A kinase activity. Consistent with our knockdown studies, applying a highly selective and potent Aurora A inhibitor, S1451, to CWR22Rv1 cells dramatically decreased AR-V7 protein levels in a dose-dependent manner without affecting FL-AR protein levels (Fig. 7a). To establish if this was a direct effect on alternative splicing, we measured FL-AR and AR-V7 transcript levels and demonstrated that S1451 treatment reduced AR-V7 transcripts in a dose-dependent fashion whilst the levels of AR-fl mRNA remained relatively unchanged (Fig. 7b). Similarly to RNAi mediated knockdown of Aurora A, kinase inhibition reduced protein levels of the splicing factor ASF/SF2 providing a mechanism of modulating AR transcripts (Fig. 7c). Consistent with our knockdown data, inhibition of Aurora A decreased expression of the AR target genes *PSA* and *TMPRSS2*, but failed to impact on *CCNA2* (Fig. 7d) suggesting an enzymatic dependency for some, but not all AR-target genes. Furthermore, S1451 significantly reduced CWR22Rv1 cell growth in a dose-dependent manner (Fig. 7e) demonstrating that AR-V-expressing PC cells are sensitive to Aurora A kinase inhibition. In all, our data provides evidence that targeting Aurora A in CRPC may be of therapeutic benefit in patients expressing distinct isoforms of the AR.

## Discussion

The AR remains a prominent target for development of novel therapeutic agents in CRPC. It has become apparent that although initially efficacious, anti-hormonal treatments select heterogeneous cell populations that utilise aberrant androgenic signalling to enable therapeutic resistance and disease progression^22^. The identification of AR isoforms that lack the ligand binding domain, termed AR-Vs, which are able to permit androgenic signalling in castrate-conditions, are not targeted by current anti-androgens providing a novel therapy escape mechanism^23^. Several lines of evidence indicate AR-Vs are key drivers of CRPC and disease progression. Consistent with this, we show that the clinically-relevant AR-V7 drives expression of a number of important cell cycle regulatory genes, including *CCNA2, CDK1* and *CDC25A*, which were significantly down-regulated upon specific AR-V7 knockdown. There is a significant clinical need therefore to develop agents that can abrogate AR-V signalling for use in patients with advanced CRPC. The N-terminal domain (NTD) of the AR is highly unstructured creating a multitude of binding surfaces for important protein interactions^24^. However, the inherent disorder of this domain has so far prevented structure-based drug design of agents that bind and attenuate AR activity via the NTD. Therefore, in order to target the activity of AR-Vs, it may provide beneficial to target upstream regulatory mechanisms to indirectly attenuate receptor variant function. Although mechanisms of AR-V regulation remain a major knowledge gap, there have been a number of recent reports describing distinct mechanisms of AR-V control, including their dependency on both pioneer factors and bromodomain and extraterminal family (BET) proteins^17^,^25^.

Aurora A has previously been identified to modulate FL-AR; by directly interacting with and phosphorylating the NTD of the receptor at positions T282 and S293, chromatin binding and transcriptional activity of the AR was shown to be enhanced. Given the sites of Aurora A-mediated phosphorylation are present in AR-Vs, we sought to establish if Aurora A also co-operates with AR-Vs to control receptor activity. In support of previous findings, we show Aurora A expression is elevated in PC, and further increased in CRPC (Fig. 1c), and is a positive regulator of FL-AR function (Supplementary Figure S1) suggesting a role in therapy-resistance and disease progression. Interestingly, expression of the *Aurora A* gene is directly regulated by AR-V-mediated signalling in CWR22Rv1 cells indicating the existence of a positive-feedback mechanism to amplify aberrant receptor function in advanced disease (Fig. 1b). In addition, we found that siRNA-mediated depletion of Aurora A reduced expression of AR-V target genes *TMPRSS2, UBE2C* and *CCNA2* in both castrate and androgen-supplemented conditions. We also observed a reduction in PSA protein levels upon depletion of Aurora A although *PSA* mRNA remained unchanged, which was surprising given the robust reduction in other AR target genes. The cause of this discrepancy between mRNA and protein levels is currently unclear but is being further investigated.

Given the effect of Aurora A knockdown on AR-V target gene expression, and evidence that the NTD of the FL-AR is directly phosphorylated by the enzyme^10^, it was unexpected that an interaction between ectopically-expressed AR-Vs and Aurora A was not detected. We speculate that in the context of FL-AR, the receptor ligand binding domain (LBD) is required for interaction with Aurora A to enable subsequent phosphorylation of the target sites within the AR NTD. The activation function-2 (AF-2) domain of the AR resides within the LBD and is responsible for mediating inter- and intra-molecular interactions with amphipathic helical LXXLL or FXXLF motifs contained within respective co-regulators and the AR NTD^26^. Interestingly Aurora A harbours an LXXLF motif (amino acid residues 161–165); mutation of which markedly reduces the interaction with FL-AR (Supplementary Figure S6) suggesting that the interaction between Aurora A and the full-length receptor may be mediated through the AF-2 domain of the AR, which is absent in AR-Vs.

We have previously shown AR-Vs to be constitutively associated with chromatin^17^, and given the role of Aurora A-mediated phosphorylation to permit recruitment of FL-AR to *cis-*regulatory elements of target genes, we hypothesised that Aurora A may enable AR-V function by facilitating chromatin deposition. To this end, ChIP and chromatin extraction methods were used to investigate the role of Aurora A in regulating global and site-specific chromatin recruitment of AR-Vs in steroid-depleted conditions in which only variant receptors would be active. Consistent with FL-AR^10^, significant reductions in recruitment of AR-Vs to chromatin was observed (Fig. 3). Critically, however, this result, together with the robust down-regulation of AR-V-mediated gene expression, was subsequently found to be a result of reduced AR-V protein levels which itself was a direct consequence of selective AR-V mRNA down-regulation (Fig. 5). This suggested a role of Aurora A in modulating the generation of different AR transcripts; a process largely distinct from that reported^10^ and observed (Supplementary Figure S1) for regulation of FL-AR. Consistent with this, using an AR-V7 minigene reporter, in which inclusion/exclusion rates of the cryptic exon 3 (CE3) of AR-V7 can be quantified directly, we demonstrated that splicing of exon3 to CE3 is markedly reduced upon Aurora A depletion. In contrast, the formation of FL-AR-like transcripts of spliced exons 3 and 4 was not compromised by down-regulated enzyme expression confirming that Aurora A discriminately regulates processing of AR pre-mRNA to generate AR-Vs in CRPC.

How Aurora A regulated this process of specific AR-V splicing was next investigated. Aurora A has previously been shown to modulate the pro-apoptotic splicing of expressed genes *Bcl-x, caspase-9* and *Mcl1* through a mechanism involving post-translational turnover of the splicing factor ASF/SF2^27^. Intriguingly, ASF/SF2 has previously been shown to regulate cryptic exon inclusion in AR pre-mRNA and hence is important for the formation of AR-V7 in CRPC cell lines^21^. Thus we hypothesised that stabilisation of ASF/SF2 by Aurora A maintains and promotes the generation of AR-V transcripts. This was confirmed by demonstrating reduced ASF/SF2 protein levels in CWR22Rv1 cells either depleted of Aurora A or grown in the presence of the Aurora kinase inhibitor S1451. The generation of AR-Vs is well studied and a number of identified mechanisms result in their synthesis, including alternative splicing and genomic deletion of exons 4–7 that encode the AR LBD^28^. In the case of the latter, genomic deletion may prove refractory to the effects of Aurora A inhibition given that the abrogation of AR-V signalling appears to be mediated principally by compromised cryptic exon inclusion and hence generation of receptor variant mRNA. However, this may not be relevant given that there is little evidence to support intragenic deletions clinically.

To determine the therapeutic potential of targeting Aurora A in CRPC, we established the phenotypic effects of Aurora A knockdown in CWR22R*v*1 cells. Aurora A depletion has previously been shown to arrest cells in the G2/M phase of the cell cycle and is consistent with a regulatory role in mitotic spindle assembly. Surprisingly, we have shown that depletion of Aurora A in the AR-positive cell lines CWR22Rv1 and LNCaP drives G1 phase arrest in addition to a modest increase in apoptosis. As a consequence of proliferation being largely AR driven in these cell lines, we attribute inhibition of AR signalling to the observed G1 cell cycle arrest in response to Aurora A depletion. This hypothesis is supported by cell cycle analysis in the AR-negative PC-3 cells that demonstrated G2/M arrest upon Aurora A knockdown. Importantly, CWR22R*v*1 cell proliferation and survival capacity was significantly diminished suggesting that targeting Aurora A is cytostatic and cytotoxic in models of advanced PC.

Selective and non-selective Aurora kinase inhibitors have entered clinical trials for a number of cancer types, including neuroendocrine prostate cancer^29^. Using the highly potent and clinically-relevant Aurora A inhibitor, S1451, we observed discriminate reductions in AR-V protein and mRNA levels, an effect not observed for FL-AR, and is in part through diminished levels of the splicing factor ASF/SF2. Treatment of CWR22Rv1 cells with S1451 also decreased AR-target gene expression and cell proliferation. These findings are consistent with our siRNA-mediated knockdown data, indicating that the enzymatic activity of Aurora A is critical for control of expression and the pro-proliferative activity of AR-Vs in this cell line. We speculate that a combination of low dose Aurora A kinase inhibitors and anti-androgens may prevent the occurrence and selection of AR-Vs to prevent or delay disease progression compared to single dose regimens. In summary, we have demonstrated diverse roles of Aurora A in the regulation of AR-V signalling providing additional support of therapeutically targeting Aurora A in advanced CRPC.

## Methods

### Gene expression profiling

*AURKA* gene expression across clinical samples was determined by analysing the pre-processed microarray dataset from the Grasso *et al*. study^19^. Gene expression values were extracted from the dataset, Accession number: GSE35988, using the Gene expression Omnibus (GEO) repository^30^. Expression values were plotted against groups based on clinical presentation (benign, localised PC and metastatic CRPC) as described in the study. Statistical testing was performed using ANOVA analysis to determine the significance of expression changes between the distinct disease groupings (GraphPad Prism 6).

### Cell culture, transfections and transductions

CWR22Rv1, LNCaP, VCaP and PC-3 cells were maintained in RPMI-1640 (Sigma Aldrich) supplemented with 10% (v/v) foetal bovine serum (Sigma Aldrich) and 2 mM L-glutamine (Sigma Aldrich) at 37 °C. For steroid-depleted conditions, cells were grown in RPMI-1640 supplemented with 10% (v/v) dextran-coated charcoal-stripped foetal bovine serum (HyClone) and 2 mM L-glutamine. Transient siRNA transfections were carried out using siRNA sequences outlined in Supplementary Table S1 using Lipofectamine RNAiMax (Thermo) according to manufacturer’s protocol. The ViraPower Lentiviral Expression system (Life Sciences) was used to generate viral particles using pLenti-AR-V7 expression vector and control pLenti-LacZ vector (Life Sciences). LNCaP cells were transfected with Aurora A siRNA or scrambled control (siScr) followed by plasmid transduction and allowed to culture for 72 hours before harvest and western blot analysis.

### Chromatin immunoprecipitation (ChIP) and chromatin extraction

Chromatin was prepared from CWR22R*v*1 cells grown in steroid-depleted conditions with 72 hour knockdown of Aurora A as described in ref. 31. ChIP was performed using 2 μg AR (N-20, Santa Cruz Biotechnology) or isotype control (Diagenode) antibodies and 80 μg prepared chromatin. DNA was extracted following ChIP and subjected to quantitative PCR using primers specific to *PSA, TMPRSS2* and *UBE2C* androgen response enhancer regions (see Supplementary Table S1). Data is presented as average fold % input (calculated as described in ref. 31) between experimental arms of at least three independent experiments. For chromatin extraction, cells were transfected for 72 hours and lysates prepared as described in ref. 32. Resultant lysates were subjected to western analysis using AR (N-20) and histone H3 (Abcam) antibodies.

### RNA extraction and qPCR, Immunoprecipitation, SDS-PAGE and Western blotting

RNA extractions were carried out as described in ref. 17 and resultant RNA was reverse transcribed using M-MLV reverse transcriptase (Promega) according to manufacturer’s instructions. AR target gene expression was analysed by quantitative PCR using synthesised cDNA and primer sequences outlined in Supplementary Table S1. Data represents a minimum of three independent experiments, statistical testing was carried out as indicated in figure legends. Immunoprecipitation was carried out as described in ref. 33 using the AR (N-20) antibody, and lysates subjected to western blot analysis as described in ref. 31 using anti AR (N-20), ASF/SF2 (96) (Santa Cruz Biotechnology), Aurora A (ab1827) (Abcam), AR-V7 (Precision Antibody), α-tubulin (Sigma) or PSA (kind gift from Kim Pettersson, University of Turku, Finland) antibodies.

### Minigene splicing

PC-3 cells were transfected with 1 μg AR-V7 minigene construct (kind gift from X. Dong, University of Vancouver, Canada) for 8 hours before re-transfection with Aurora A targeting siRNA or Scr control and cultured for an additional further 48 hours. Cells were harvested, RNA extracted and resultant cDNA subjected to qPCR as described using specific FL-AR and AR-V7 primers (Supplementary Table S1).

### Cell proliferation, colony forming and flow cytometry analysis

Cell growth was measured using the IncucyteZoom live cell imager (Essen Bioscience) as a measurement of % confluency. CWR22Rv1 cells were grown in steroid-depleted conditions −/+ DHT (10 nM) or enzalutamide (10 μM) following Scr control or Aurora A targeting siRNA transfections. Images were collected from multiple fields every 6 hours over a 96 hour period and % confluence data analysed. Cell confluence was normalised for time point 0 and presented as relative fold change to the siScr experimental arm. Data represents at least three independent experiments −/+ SEM. For colony forming assays, CWR22R*v*1 cells were transfected with siRNA on 90-mm dishes for 48 hours before re-seeding at a density of 200 cells/well of a 6-well plate. Cells were allowed to adhere and were cultured for 12 days with replenishment of media every 3–4 days. Cells were fixed using fixative solution (acetic acid: methanol 3:1) and stained with crystal violet. Presented data represents the average of three independent experiments where colonies were counted and normalised as a percentage of the siScr experimental arm. Propidium iodide staining was used to measure percentage of cells in distinct cell cycle phases by flow cytometry. siRNA transfected CWR22R*v*1 cells were cultured for 72 hours in steroid-depleted conditions prior to cell preparation, as described in ref. 33. Propidium iodide signal was detected using a FACSCalibur (BD Biosciences), and data analysed using Cyflogic software (CyFlo Ltd.). Data presented represents the mean percentage of cells in each cell cycle phase over three independent experiments −/+ SEM.

## Additional Information

**How to cite this article**: Jones, D. *et al*. Aurora A regulates expression of AR-V7 in models of castrate resistant prostate cancer. *Sci. Rep.* **7**, 40957; doi: 10.1038/srep40957 (2017).

**Publisher's note:** Springer Nature remains neutral with regard to jurisdictional claims in published maps and institutional affiliations.

## Supplementary Material

Supplementary Information
