# Supplementary material for: Aurora A regulates expression of AR-V7 in models of castrate resistant prostate cancer
Source: Sci Rep. 2017 Feb 16;7:40957. doi: 10.1038/srep40957 (PMC5311967; doi:10.1038/srep40957)
Supplement: Supplementary Information [file srep40957-s1.pdf]

Supplementary information

Aurora A regulates expression of AR-V7 in models of castrate resistant prostate cancer.

Dominic Jones, Martin EM Noble, Steve R Wedge, Craig N Robson and Luke Gaughan

a.

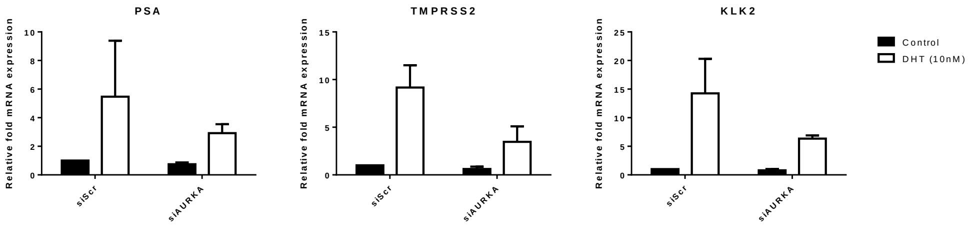

b.

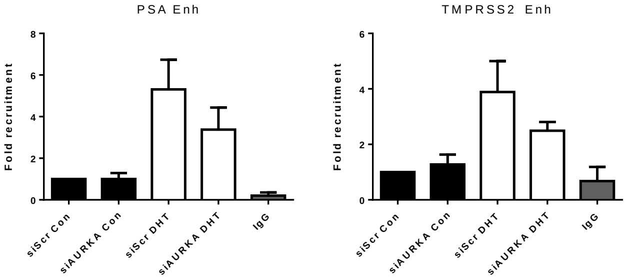

Supplementary Figure S1

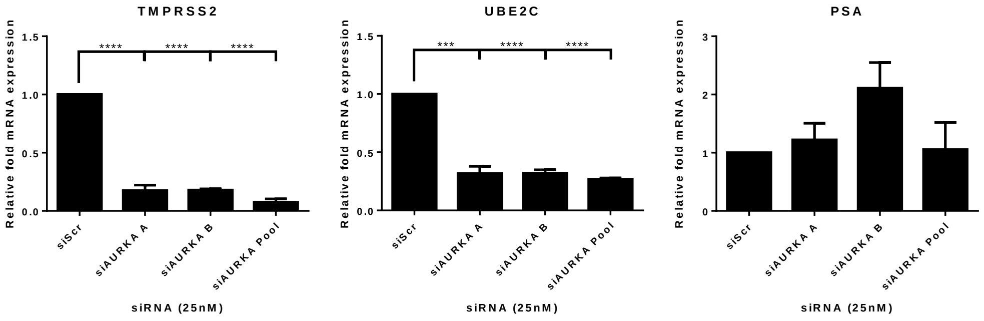

Supplementary Figure S2

LNCaP

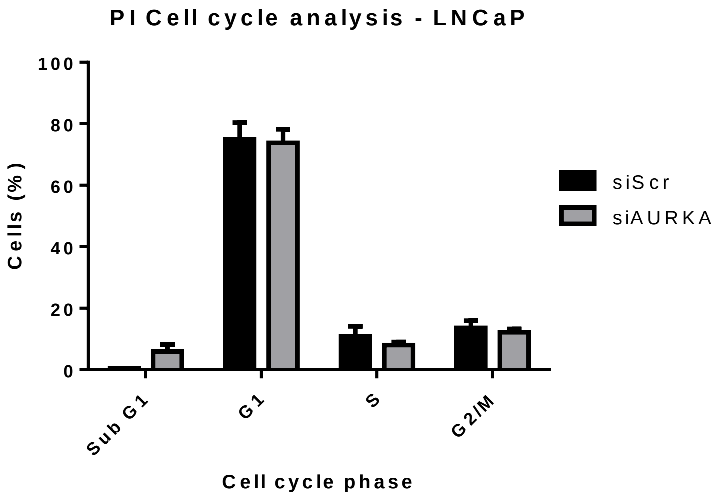

PC-3 cells

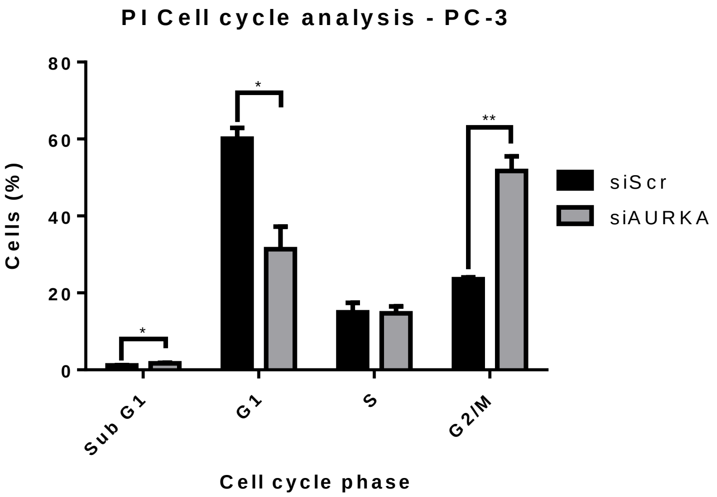

Supplementary Figure S3

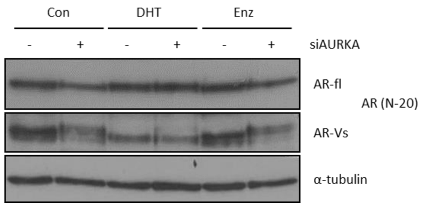

Supplementary Figure S4

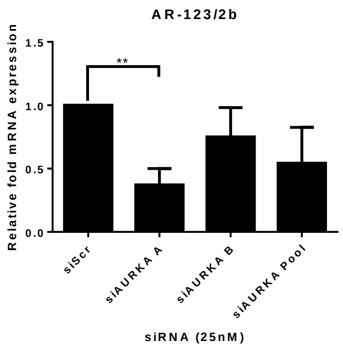

Supplementary Figure S5

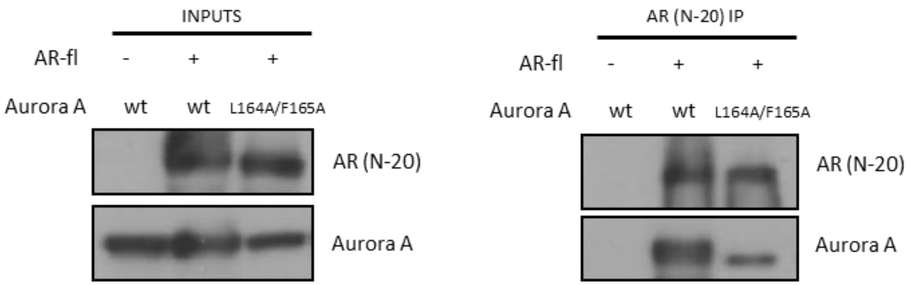

Supplementary Figure S6

<u>Supplementary information to accompany Jones *et al*.,</u>

Supplementary Table S1. siRNA and primer sequences

Oligo Name	Sequence (5’-3’)
**PSA mRNA F**	GCAGCATTGAACCAGAGGAG
**PSA mRNA R**	AGAACTGGGGAGGCTTGAG
**TMPRSS2 mRNA F**	CTGCTGGATTTCCGGGTG
**TMPRSS2 mRNA R**	TTCTGAGGTCTTCCCTTTCTCCT
**UBE2C mRNA F**	TGCCCTGTATGATGTCAGGA
**UBE2C mRNA R**	GGGACTATCAATGTTGGGTTCT
**CCNA2 mRNA F**	GAAGACGAGACGGGTTGCA
**CCNA2 mRNA R**	AGGAGGAACGGTGACATGCT
**CDC25A mRNA F**	CAAACCTTGACAACCGATGC
**CDC25A mRNA R**	ACACTGACCGAGTCGTGGAG
**CDK1 mRNA F**	CCTAGCATCCCATGTCAAAAACTTGG
**CDK1 mRNA R**	TGATTCAGTGCCATTTTGCCAGA
**AURKA mRNA F**	GGGTGGTCAGTACATGCTCC
**AURKA mRNA R**	AGGCTCCAGAGATCCACCTT
**AR-fl mRNA F**	AAGAGAAGTACCTGTGCGCC
**AR-fl mRNA R**	TTCAGATTACCAAGTTTCTTCAG
**AR-V7 mRNA F**	AAGAGAAGTACCTGTGCGCC
**AR-V7 mRNA R**	TCAGGGTCTGGTCATTTTGA
**AR-123/2b mRNA F**	AAGAGAAGTACCTGTGCGCC
**AR-123/2b mRNA R**	TTCTGTCAGTCCCATTGGTG
**HPRT1 mRNA F**	TTGCTTTCCTTGGTCAGGCA
**HPRT1 mRNA R**	AGCTTGCGACCTTGACCATCT

Oligo Name	Sequence (5'-3')
**siARexCe3**	GUAGUUGUGAGUAUCAUGA
**siARex1**	CAAGGGAGGUUACACCAAA
**siAURKA A**	AUGCCCUGUCUUACUGUCA
**siAURKA B**	GGCCAAUGCUCAGAGAAGUA
**siScrambled**	UUCUCCGAACGUGUCACGU

Oligo Name	Sequence (5'-3')
**PSA Enh ChIP F**	TGGGACAACTTGCAAACCTG
**PSA Enh ChIP R**	CCAGAGTAGGTCTGTTTTCAATCCA
**TMPRSS2 Enh ChIP F**	TGGTCCTGGATGATAAAAAAAGTT
**TMPRSS2 Enh ChIP R**	GACATACGCCCCACAACAGA
**UBE2C Enh ChIP F**	TGCCTCTGAGTAGGAACAGGTAAGT
**UBE2C Enh ChIP R**	TGCTTTTTCCATCATGGCAG

Supplementary figure legends

Supplementary Figure S1. A. LNCaP cells grown in steroid-depleted media were subject to Aurora A knockdown by siRNA for 48 hours before treatment with 10 nM DHT for a further 24 hours and then harvested. *PSA TMPRSS2* and *KLK2* expression was measured by quantitative PCR from resultant cDNA. **B.** Chromatin immunoprecipitation (ChIP) was carried out using the AR (N-20) antibody in LNCaP cells depleted of Aurora A kinase for 46 hours and treated with 10 nM DHT for a further 2 hours before harvesting.

Supplementary Figure S2. CWR22R*v*1 cells were transiently transfected with two individual siRNAs targeting Aurora A (siAURKA A and siAURKA B) and a combination of the two (siAURKA Pool) for 72 hours before *TMPRSS2, UBE2C* and *PSA* expression analysis by quantitative PCR.

Supplementary Figure S3. LNCaP (left panel) and PC-3 (right panel) cells were subjected to propidium iodide flow cytometry following 72 hour knockdown of Aurora A. % of cells in G2/M phase of the cell cycle is shown.

Supplementary Figure S4. VCaP cells grown in steroid-depleted media were transiently transfected with Aurora A siRNA for 48 hours prior to 24 hour treatment with 10 nM DHT or 10 µM enzalutamide and western analysis using anti-AR and –α-tubulin antibodies.

Supplementary Figure S5. Expression of AR-123/2b mRNA was measured by quantitative PCR in CWR22R*v*1 cells grown in steroid-depleted conditions and transiently transfected with Aurora A siRNAs for 72 hours.

Supplementary Figure S6. Co-immunoprecipitation was carried out using the AR (N-20) antibody on HEK293 cells overexpressing either wild type Aurora A (wt) or Aurora $A_{L164A/F165A}$ and FL-AR followed by subsequent western analysis using anti-AR (N-20) and –Aurora A antibodies. Input samples were also subjected to western blot analysis (right panel).
